# Malignant Epithelioid Soft Tissue Tumours- A Pathologist's Perspective With Review of Literature

**DOI:** 10.7759/cureus.12263

**Published:** 2020-12-24

**Authors:** Biswajit Dey, Bheemanathi Hanuman Srinivas, Bhawana Badhe, Rajesh Nachiappa Ganesh, Debasis Gochhait, Pampa C Toi, Sreerekha Jinkala

**Affiliations:** 1 Pathology, North Eastern Indira Gandhi Regional Institute of Health and Medical Sciences, Shillong, IND; 2 Pathology, Jawaharlal Institute of Postgraduate Medical Education and Research, Puducherry, IND

**Keywords:** sarcoma, immunohistochemistry, pathologists

## Abstract

Background

Soft tissue tumours with epithelioid morphology have many differential diagnoses, which include epithelioid sarcoma, malignant extrarenal rhabdoid tumour, epithelioid malignant peripheral nerve sheath tumour, epithelioid leiomyosarcoma, epithelioid angiosarcoma and sclerosing epithelioid fibrosarcoma. There are other rare entities also. They often express characteristic patterns of immunohistochemical markers that can be used to identify these tumours.

Materials and methods

This retrospective study comprises 22 cases of malignant soft tissue tumours with epithelioid differentiation diagnosed over a period of four years. Findings regarding clinical presentation, cytological findings, histopathological findings and immunohistochemical profile of the tumours were noted and analyzed.

Results

A total of 22 cases were included in the study including five cases of epithelioid sarcoma (conventional and proximal), three cases of epithelioid angiosarcoma and epithelioid myxofibrosarcoma, two cases of epithelioid malignant peripheral nerve sheath tumour, epithelioid gastrointestinal stromal tumour and clear cell sarcoma each, one case of epithelioid leiomyosarcoma, sclerosing epithelioid fibrosarcoma, malignant extrarenal rhabdoid tumour, monophasic synovial sarcoma and malignant and malignant perivascular epithelioid cell tumour each.

Conclusion

Pathologists should be aware of the various differential diagnoses of soft tissue tumours with epithelioid morphology. Over and above the clinical findings and morphological features, ancillary methods like immunohistochemistry help to arrive at a definitive diagnosis in most cases.

## Introduction

Epithelioid morphology is a key feature of several soft tissue tumours and has been described in various benign and malignant tumours. Malignant epithelioid soft tissue tumours are morphologically distinct malignant neoplasms which include epithelioid sarcoma (ES), malignant extrarenal rhabdoid tumour (MERT), epithelioid malignant peripheral nerve sheath tumour (EMPNST), epithelioid leiomyosarcoma, epithelioid angiosarcoma and sclerosing epithelioid fibrosarcoma (SEF) [1]. The other rare entities, where epithelioid variant have been described are epithelioid myxofibrosarcoma, epithelioid gastrointestinal stromal tumour, epithelioid rhabdomyosarcoma, epithelioid pleomorphic liposarcoma and epithelioid inflammatory myofibroblastic sarcoma [1,2]. Clear cell sarcoma, solid alveolar soft part sarcoma, monophasic synovial sarcoma and malignant perivascular epithelioid cell tumour (PEComa) are also described to exhibit epithelioid morphology [2].

The chief significance of these neoplasms is that they morphologically resemble true epithelioid tumours, in particular carcinoma. Therefore, the study of the histological features alone can leave the pathologist with a long list of differential diagnoses. However, malignant epithelioid soft tissue tumours often express characteristic patterns of immunohistochemistry (IHC) markers that can be used to identify these tumours [1,2]. Thus, morphology and IHC along with clinical features are pivotal in arriving at a definitive diagnosis.

The present study was undertaken to study the various soft tissue tumours with epithelioid morphology and their immunohistochemical characteristics diagnosed in a tertiary care institute.

## Materials and methods

This retrospective study comprises 22 cases of malignant soft tissue tumours with epithelioid differentiation diagnosed over a period of four years from January 2011 to December 2014 in a tertiary care institute. A detailed clinicopathological and immunohistochemical profile of the tumours were noted.

Preoperative fine-needle aspiration biopsy (FNAB) was performed in seven cases. Slides were stained for May-Grunwald Giemsa (MGG) and Papanicolaou (Pap) stains. All cases were documented by preoperative core needle biopsy. All the samples were fixed in 10% neutral buffered formalin and embedded in paraffin. Staining was done with haematoxylin and eosin. IHC staining for different monoclonal antibodies was done by Avidin-Biotin peroxidase method with pre-treatment by microwave heating.

## Results

A total of 22 cases were included in the study. These include five cases of epithelioid sarcoma (conventional and proximal), three cases of epithelioid angiosarcoma and epithelioid myxofibrosarcoma, two cases of epithelioid malignant peripheral nerve sheath tumour (MPNST), epithelioid gastrointestinal stromal tumour (GIST) and clear cell sarcoma each, one case of epithelioid leiomyosarcoma, SEF, MERT, monophasic synovial sarcoma and malignant PEComa each. The clinical findings and immunohistochemistry done in these cases are summarized in Table 1.

**Table 1:** Summary of clinical features and immunohistochemistry of the 22 cases of soft tissue tumours with epithelioid morphology IHC: Immunohistochemistry; INI 1: Integrase interactor 1; EMA: Epithelial membrane antigen; HMB-45: Human melanoma black; DOG1: Discovered on GIST1

Diagnosis	Number of cases (n=22)	Age (years)	Site	Size (cm)	Positive IHC	Negative IHC
Epithelioid Sarcoma (Conventional)	3	27 to 47	Lower extremities	5 to 25	Vimentin, cytokeratin and CD34	S100, CD99, desmin, smooth muscle actin and INI 1
Epithelioid Sarcoma (Proximal type)	2		Upper extremities			
Malignant Extrarenal Rhabdoid Tumour	1	35	Scapula	4	Vimentin, cytokeratin and CD99	S100, desmin, CD34 and INI 1
Epithelioid malignant peripheral nerve sheath tumour	2	63 and 50	Chest and left leg	6 and 12	S-100 and vimentin	Cytokeratin, EMA, desmin, CD34, Melan-A and HMB-45
Epithelioid Leiomyosarcoma	1	50	Left leg		Smooth muscle actin and vimentin	Cytokeratin, CD34, S100, HMB-45 and CD117
Epithelioid angiosarcoma	3	40 to 65	Lower extremities	15 to 25	CD31 and factor VIII	Cytokeratin, EMA and desmin
Sclerosing epithelioid fibrosarcoma	1	50	Left knee	12	Vimentin	Cytokeratin, EMA, HMB45, desmin and alpha-smooth muscle actin
Epithelioid myxofibrosarcoma	3	43 to 45	Lower extremities	10 to 20	-	All negative
Epithelioid gastrointestinal stromal tumour	2	48 and 58	Stomach and ileum	12 and 21	CD117 and DOG1	Cytokeratin, EMA, HMB45, desmin and alpha-smooth muscle actin
Clear cell sarcoma	2	17 and 35	Left thigh and chest wall	10 and 6	HMB-45, neuron-specific enolase and S-100	Cytokeratin, EMA, carcinoembryonic antigen, desmin, Melan-A and smooth muscle actin
Monophasic synovial sarcoma	1	50	Inguinal region	5	Cytokeratin, EMA and vimentin	Smooth muscle actin, S100 and CD117
Malignant perivascular epithelioid cell tumour	1	50	Retroperitoneum	8	HMB45, Melan-A and actin	Cytokeratin, S100 and CD117

Epithelioid sarcoma and malignant extrarenal rhabdoid tumour

There were five cases of epithelioid sarcoma, out of which two were conventional epithelioid sarcoma and three were proximal epithelioid sarcoma. The age of the patients ranged from 27 to 47 years. The sites of both the conventional epithelioid sarcoma were lower extremities whereas the sites of proximal epithelioid sarcoma were proximal part of upper extremities. The size of the tumours varied from 5 to 25 cm in the largest dimension.

**Figure 1:**
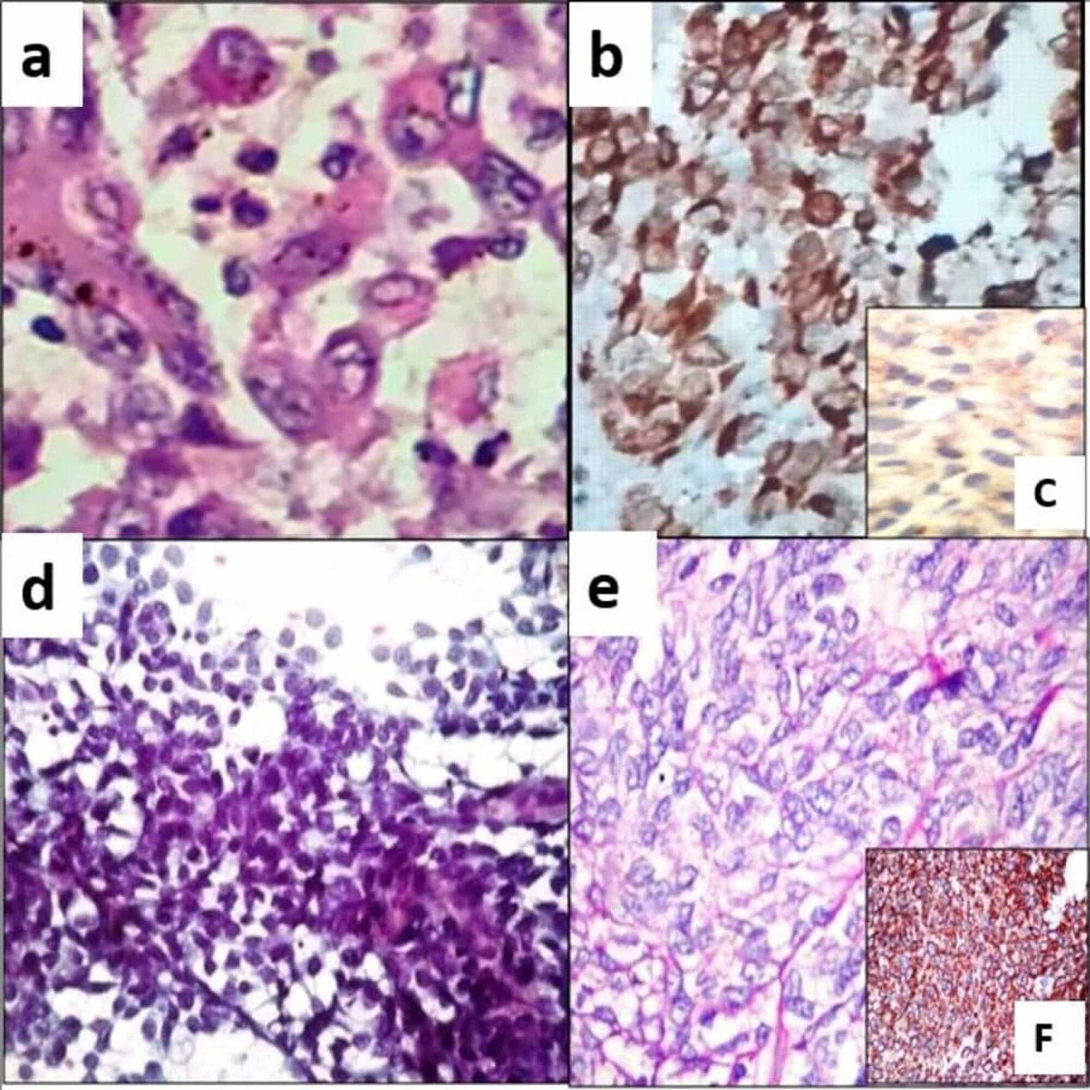
Epithelioid sarcoma (a) Histopathology showed tumour cells with epithelioid differentiation with prominent nucleoli and a moderate amount of eosinophilic cytoplasm. (H & E, 400x); (b) Tumours cells are positive for vimentin (IHC, 400x); (c) Loss of INI 1 in the tumour cells (IHC, 200x) Epithelioid malignant peripheral nerve sheath tumour (d) Smears showed pseudopapillaroid fragments of polygonal to spindly malignant cells (Pap, 100x); (e) Histopathology showed epithelioid cells surrounded by fibrous stroma (H & E, 400x); (f) Tumour cells are positive for S100 (IHC, 200x) H & E: Hematoxylin and eosin; IHC: Immunohistochemistry; Pap: Papanicolaou

FNAB done in one case showed scattered and loosely cohesive clusters of pleomorphic cells with vesicular nuclei and a moderate amount of cytoplasm, which on histopathology was diagnosed as proximal epithelioid sarcoma. On histopathology, all the tumours showed geographic areas of necrosis with palisading tumour cells with epithelioid differentiation with prominent nucleoli and a moderate amount of eosinophilic cytoplasm (Figure 1a). Mitotic count in conventional ES ranged from 8-10/10 high power field (HPF), whereas in proximal ES mitosis ranged from 10-15/10 HPF with atypical mitosis. On IHC, tumour cells were positive for vimentin, cytokeratin and CD34 (Figure 1b). The negative immunomarkers were S100, CD99, desmin, smooth muscle actin and Integrase interactor 1 (INI 1) (Figure 1c).

There was one case of MERT in a 35-year-old male presented with a scapular swelling measuring 4 cm in the largest dimension. Histologically it showed rhabdoid cells with eccentric vesicular nuclei, prominent nucleoli, and abundant cytoplasm. On IHC, tumour cells were positive for vimentin, cytokeratin, and CD99. S100, desmin, CD34, and INI 1 were negative.

Epithelioid malignant peripheral nerve sheath tumour (EMPNST)

There were two cases of EMPNST. One patient was a 63-year-old male, who presented with a painful chest wall swelling measuring 6 cm in the largest dimension and the other patient was a 50-year-old male, who presented with a left leg swelling measuring 12 cm in the largest dimension. FNAB was done in both the cases and smears showed three-dimensional pseudopapillaroid fragments, as well as dissociated polygonal to spindly malignant cells (Figure 1d). Histopathology showed epithelioid cells surrounded by fibrous stroma (Figure 1e). Mitosis ranged from 10-15 HPF. On IHC, tumour cells were positive for S-100 and vimentin, while negative for cytokeratin, epithelial membrane antigen (EMA), desmin, CD34, Melan-A, and human melanoma black (HMB-45) (Figure 1f).

Epithelioid leiomyosarcoma

There was one case of epithelioid leiomyosarcoma of the left leg in a 50-year-old male. Histopathology showed round to polygonal cells with vesicular nuclei and slightly eosinophilic cytoplasm arranged in sheets. Mitosis was 6/10 HPF. Positive immunoprofile consisted of smooth muscle actin and vimentin. Negative markers were cytokeratin, CD34, S100, HMB-45, and CD117. 

Epithelioid angiosarcoma

There were three cases of epithelioid angiosarcoma, all located in the lower extremities with sizes varying from 15 to 25 cm. The age of the patients ranged from 40 to 65 years. Histopathology showed epithelioid morphology with prominent small-to-medium-sized vessels. Haemorrhage and necrosis noted in all the cases. Mitotic count ranged from 10 to 20/HPF. Immunoprofile for CD31 and factor VIII was positive. Tumour cells were negative for cytokeratin, EMA, and desmin.

Sclerosing epithelioid fibrosarcoma (SEF)

**Figure 2:**
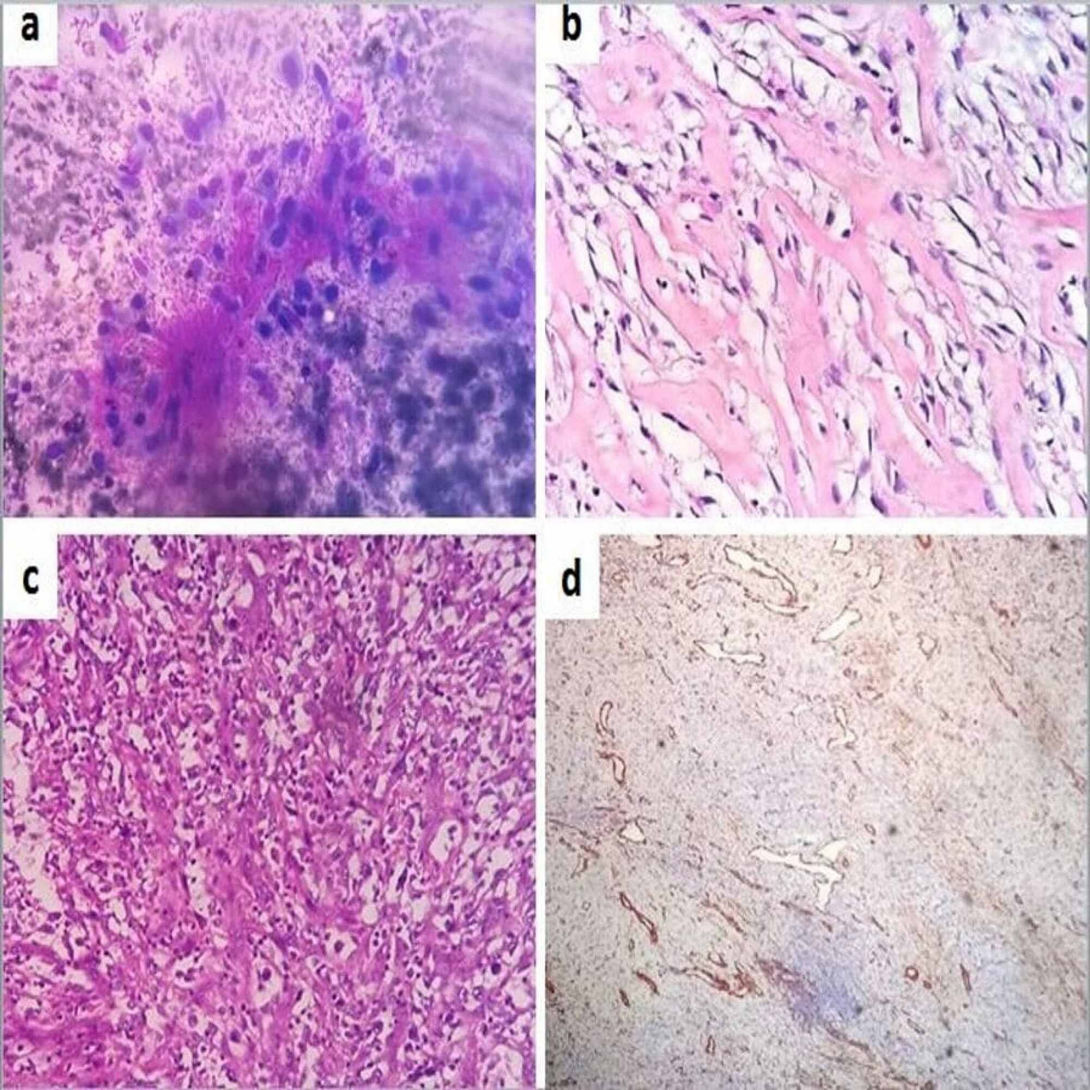
Sclerosing epithelioid fibrosarcoma (a) Smears showed ovoid epithelioid cells embedded in a collagenous matrix (MGG, 200x); (b) Histopathology showed small, slightly angulated, round to ovoid epithelioid cells with sparse cytoplasm along with prominent hyaline sclerosis (H & E, 400x); Epithelioid myxofibrosarcoma (c) Histopathology showed hypercellular area consisting of epithelioid cells arranged singly and in small clusters (H & E, 200x); (d) All immunomarkers were negative (IHC, 40x) MGG: May-Grunwald Giemsa; H & E: Hematoxylin and eosin; IHC: Immunohistochemistry

There was one case of SEF in a 50-year-old male, who presented with a left knee swelling measuring 12 cm in the largest dimension. FNAB showed ovoid epithelioid cells embedded in a collagenous matrix (Figure 2a). Histologically there was a proliferation of small, slightly angulated, round to ovoid epithelioid cells with sparse, often clear cytoplasm along with prominent hyaline sclerosis (Figure 2b). Mitosis was sparse. Vimentin was positive immunohistochemically. Cytokeratin, EMA, HMB45, desmin, and alpha-smooth muscle actin were negative.

Epithelioid myxofibrosarcoma

There were three cases of epithelioid myxofibrosarcoma, all located in the lower extremities with sizes varying from 10 to 20 cm. The age of the patients ranged from 43 to 45 years. On histopathology, all the cases were characterized by hypercellular and hypocellular myxoid areas. Tumour cells with epithelioid morphology were arranged singly and in small clusters in the hypercellular areas (Figure 2c). Prominent curvilinear vessels are seen in the hypocellular areas. All the cases were negative for all immunomarkers (Figure 2d).

Epithelioid gastrointestinal stromal tumour

**Figure 3:**
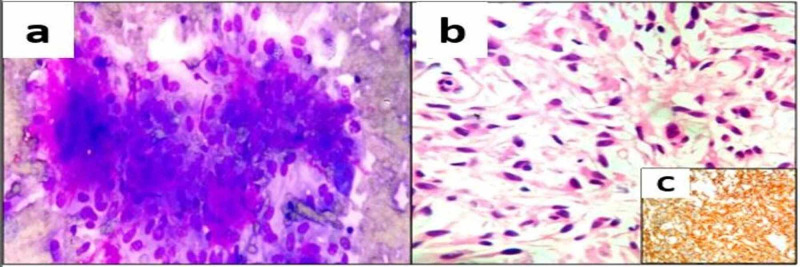
Epithelioid gastrointestinal stromal tumour (a) Smears showed loosely cohesive clusters of epithelioid cells with skenoid fibres (MGG, 200x); (b) Histopathology showed oval to rounded cells arranged in nests or sheets, with variably eosinophilic to clear cytoplasm (H & E, 200x); (c) Tumour cells positive for CD117 (IHC, 200x) MGG: May-Grunwald Giemsa; H & E: Hematoxylin and eosin; IHC: Immunohistochemistry

There were two cases of epithelioid GISTs. One patient was a 48-year-old male, who presented with a gastric mass with perforation measuring 12 cm in maximum dimension and the other patient was a 58-year-old male, who presented with an ileal mass measuring 21 cm in maximum dimension. Ileal mass was subjected to FNAB, which showed loosely cohesive clusters of cells with epithelioid differentiation with increased vascularity and collagen-like material representing skenoid fibres (Figure 3a). On histopathology both the cases showed rounded cells arranged in nests or sheets, with variably eosinophilic to clear cytoplasm and vesicular nuclei (Figure 3b). Immunohistochemically, tumour cells were positive for CD117 and Discovered on GIST1 (DOG1) (Figure 3c).

Clear cell sarcoma

**Figure 4:**
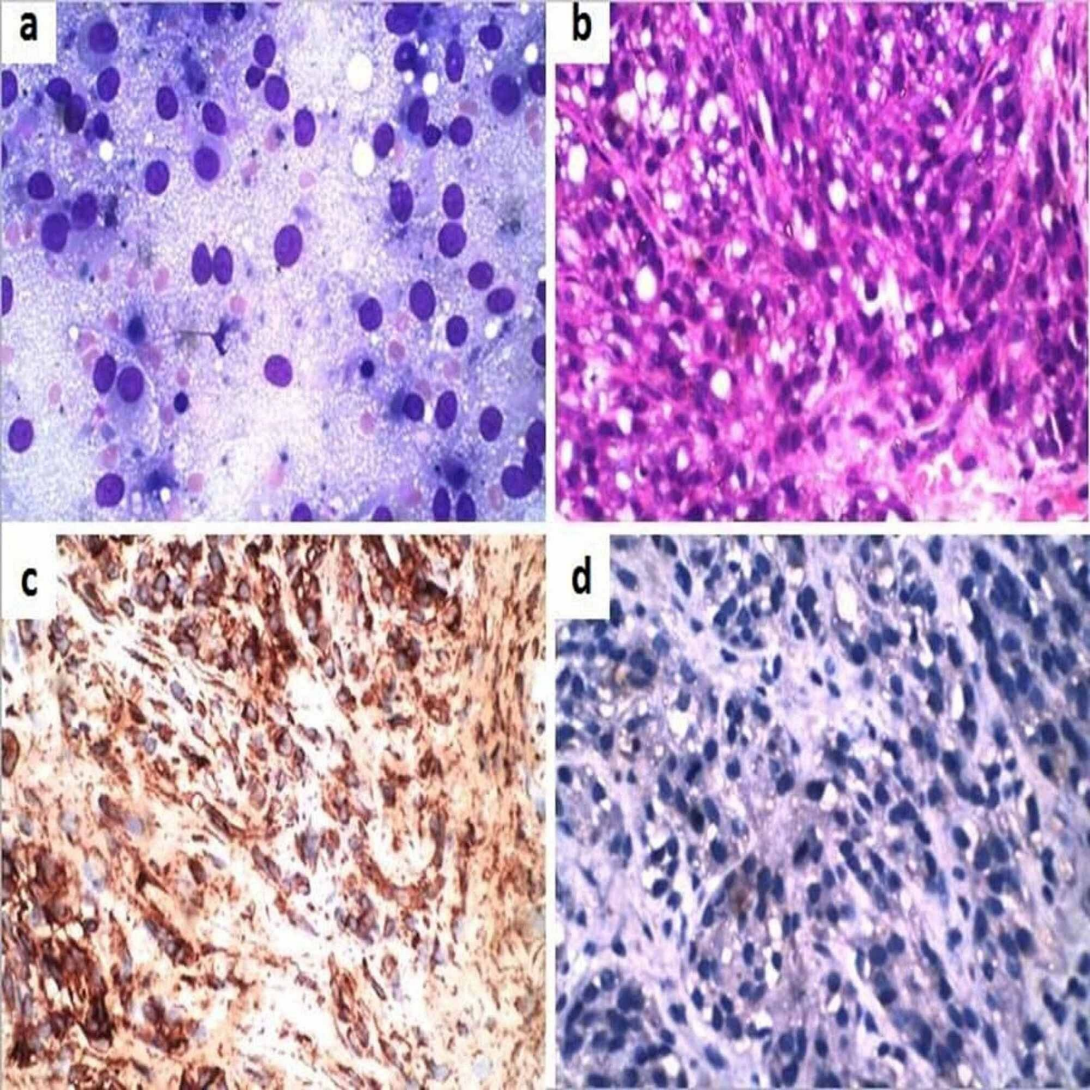
Clear cell sarcoma (a) Smears showed large polygonal cells with abundant wispy cytoplasm, round to oval nuclei, and prominent nucleoli (MGG, 400x); (b) Histopathology showed polygonal to spindle-shaped cells with clear to eosinophilic cytoplasm arranged in fascicles. (H & E, 200x); (c) Tumour cells were positive for HMB 45 (IHC, 200x); (d) Tumour cells negative for cytokeratin (IHC, 200x) MGG: May-Grunwald Giemsa; H & E: Hematoxylin and eosin; IHC: Immunohistochemistry

There were two cases of clear cell sarcoma. One of the patients was a 17-year-old female who presented with a left thigh mass measuring 10 cm in greatest dimension. The other patient was a 35-year-old male with a lateral chest wall mass measuring 6 cm in greatest dimension. Radiologically the first case was noted within the vastus medialis and adductor muscles without any bony involvement. The second case was seen arising in the pectoralis muscle without any involvement of the lung, pleura, or ribs. Both the cases were diagnosed as clear cell sarcoma in pre-operative FNAB. Cytologically there were large polygonal cells with abundant wispy cytoplasm, round to oval nuclei, and prominent nucleoli (Figure 4a). The post-operative histomorphology showed polygonal to spindle-shaped cells arranged in fascicles. The tumour cells had moderate to abundant clear to eosinophilic cytoplasm and prominent eosinophilic nucleoli (Figure 4b). No melanin pigment noted. On IHC, the tumour cells were positive for HMB-45, neuron-specific enolase, and S-100. Cytokeratin, EMA, carcinoembryonic antigen, desmin, Melan-A, and smooth muscle actin were negative (Figure 4c, 4d).

Monophasic synovial sarcoma

There was one case of monophasic synovial sarcoma in a 50-year-old male, who presented with a hard inguinal mass measuring 5 cm in greatest dimension. Preoperative trucut biopsy revealed a highly cellular tumour composed of monomorphic plump spindled shaped cells arranged in short fascicles. The tumour cells had ovoid nuclei and minimal cytoplasm. Mitoses were frequent. On IHC, the tumour cells were positive for cytokeratin, EMA, and vimentin. Smooth muscle actin, S100, and CD117 were negative. The post-operative sample was not available as the patient was lost to follow-up.

Malignant perivascular epithelioid cell tumour (PEComa)

There was a single case of malignant PEComa in a 50-year-old male. The patient had a retroperitoneal mass measuring 8 cm in maximum dimension. Preoperative FNAC showed scant with occasional pleomorphic cells with vesicular nuclei. The mass was resected and histopathology showed tumour cells with abundant clear to fine eosinophilic granular cytoplasm and round to oval nuclei with dispersed chromatin and prominent nucleoli arranged in nests or wide fascicles with delicate vascular septa. The mitotic rate was 2/50 HPF with focal areas of necrosis. On IHC, tumour cells were positive for HMB45, Melan-A, and actin. Cytokeratin, S100, and CD117 were negative.

## Discussion

Neoplasms with epithelioid morphology are usually carcinomas. However, some sarcomas demonstrate epithelioid morphology [3]. The list of such tumours is exhaustive. Despite the fact that epithelioid change might be found in a wide assortment of sarcomas, this morphology is generally characteristic for ES, MERT, EMPNST, and SEF [1].

Epithelioid sarcoma

ES is a rare soft tissue sarcoma first described by Enzinger in 1970 [4]. It typically occurs in the distal extremities of young adults as small subcutaneous nodules [1]. In 1997, a more aggressive form arising from more proximal locations was described. In contrast to conventional ES, proximal ES occurs as large nonspecific soft tissue masses [5]. It is more common in males than in females. The most common affected sites are the hands and fingers, followed by the wrist and lower arm, and lower leg and knee [1]. However, conventional ES can occur in any location [1]. The involvement of tendons and aponeuroses is common. Proximal ES tends to occur in older adults, most often involving the deep soft tissues of the perineum, genital region, and pelvic soft tissues [5].

Histologically, the conventional ES is composed of spindle-to-polygonal epithelioid cells arranged in nodules that commonly exhibit central necrosis. It consists of large epithelioid carcinoma-like and/or rhabdoid cells [6]. The cells of proximal ES show a greater degree of pleomorphism with more frequently encountered mitosis [1]. ES is immunopositive for vimentin and for a variety of low and high molecular cytokeratins except for cytokeratins 5/6. Around 50% of cases are positive for CD34 [1]. Over 90% of conventional and proximal ES show the loss of expression of the tumour-suppressor gene product INI1 (SMARCB1) [6].

ES shows substantial overlap with MERT in terms of histomorphology, immunohistochemical features, and molecular genetics [1].

Malignant extrarenal rhabdoid tumour (MERT)

The term ‘MERT’ is applied to primitive malignant tumours indicating rhabdoid histomorphology even if it is partial, loss of SMARCB1 protein and/or SMARC1 gene mutation, and without any other line of differentiation [1]. MERT has aggressive behaviour and usually involves deep axial locations like the paraspinal region and the neck. It usually occurs in infants and young children, although rare cases have been described in adults [7].

Grossly, MERT is usually less than 5 cm in dimension and is soft fleshy in consistency, grey to tan in colour, and has hemorrhagic/necrotic areas. Histologically it shows cells with ‘rhabdoid’ morphology with eccentrically placed vesicular nuclei, prominent nucleoli, and abundant cytoplasm [1]. The cytoplasm of these 'rhabdoid' cells has perinuclear hyaline inclusions or globules, which are PAS-positive [1,7]. Vimentin is almost always immunopositive in MERT and cytokeratin is usually immunopositive [1,7]. Immunoreactivity for INI1 is consistently lost in MERT [1]. Other markers such as EMA, smooth muscle actin, CD99, CD57, synaptophysin, and S100 protein may occasionally show immunopositivity [7].

Epithelioid malignant peripheral nerve sheath tumour (EMPNST)

EMPNST represents under 5% of all malignant peripheral nerve sheath tumours (MPNSTs). EMPNST most commonly occurs in the age group of 20-50 years with a male preponderance [1]. Major nerves are commonly involved by EMPNST with lower extremities and trunk being the most frequently affected anatomical sites [1,8]. Unlike the typical MPNST, EMPNST has an infrequent association with neurofibromatosis 1 and occasional origin in a schwannoma [8]. EMPNSTs behave aggressively with approximately 50% of patients ending up with distant metastases [1].

Microscopically, tumours are comprised of a relatively uniform but clearly atypical population of epithelioid cells. The majority of tumours demonstrated a multilobular growth pattern, with lobules and nests surrounded by myxoid and/or fibrous stroma [8]. Mitotic activity is invariably present and necrosis is frequently seen. In contrast to typical MPNSTs, which have a patchy and weak immunoexpression of S100, EMPNST shows strong and diffuse immunopositivity for S100 [1]. Melanocytic markers such as HMB45 and Melan-A are negative in EMPNST and this fact helps to rule out its closest differential diagnosis of amelanotic melanoma. Cytokeratin is consistently negative [8]. Approximately half of the cases of EMPNSTs show loss of INI1 expression [1].

Epithelioid leiomyosarcoma

Epithelioid leiomyosarcoma is most commonly encountered in the uterus and is rarely reported in soft tissue [9]. The tumour behaviour of epithelioid leiomyosarcomas arising from deep external soft tissues is not known [10]. The tumour is mainly composed of sheets of round to polygonal cells. The cells have mildly eosinophilic cytoplasm with a round to oval vesicular nuclei and multiple prominent nucleoli [9,10]. Mitotic figures are brisk. Immunohistochemically, it shows positive immunoreactivity for smooth muscle actin and h-caldesmon but does not express desmin. It is also negative for cytokeratin, EMA, HMB-45, CD117, and CD34. The closest differential diagnosis is epithelioid GIST, which is positive for CD117 and DOG1 [10].

Epithelioid angiosarcoma

Epithelioid angiosarcoma is a highly aggressive endothelial cell malignancy, most often arises in the deep soft tissues of the extremities [11]. It usually occurs in adult life, with the highest incidence in the seventh decade [12]. Microscopically it shows sheets of large, mildly to moderately pleomorphic epithelioid cells with abundant eosinophilic cytoplasm, vesicular nuclei, and prominent nucleoli [11,12]. Occasional cells with intracytoplasmic lumina containing erythrocytes can be identified. Mitotic figures are numerous, and varying degrees of necrosis and hemorrhage are present [11]. Epithelioid angiosarcoma is strongly vimentin and factor VIII positive [12]. CD31 is a sensitive marker, being at least weakly positive in almost all cases. CD34 positivity ranges from 40% to 100% [11]. Pancytokeratin positivity ranges from 78% to 100% in the epithelioid angiosarcoma [12].

The important differential diagnosis is epithelioid sarcoma, especially pseudoangiosarcomatous growth pattern described in proximal type epithelioid sarcoma. Immunoreactivity for endothelial markers, such as CD31, erythroblast transformation-specific-related gene (ERG), and von Willebrand factor (vWF) helps to differentiate epithelioid angiosarcoma from epithelioid sarcoma [13].

Sclerosing epithelioid fibrosarcoma (SEF)

SEF occurs primarily in the deep musculature of the extremities in adults with a high metastatic rate. Morphologically it is characterized by a proliferation of rather uniform, small, slightly angulated, round to ovoid epithelioid cells with sparse, often clear cytoplasm arranged in distinct nests and cords. Prominent hyaline sclerosis, sometimes reminiscent of osteoid or cartilage and foci of conventional fibrosarcoma is characteristic. Mitosis is generally sparse. Immunohistochemically, SEF is consistently positive for vimentin. EMA and cytokeratin may be positive in a small percentage of cases. HMB45, desmin, and alpha-smooth muscle actin are negatively expressed [14].

Epithelioid myxofibrosarcoma

The myxoid type of malignant fibrous histiocytoma (MFH), also known as myxofibrosarcoma (MFS), is the second most frequent subtype of MFH with aggressive behaviour [15]. It occurs in the elderly and has a predilection for the limbs. Epithelioid MFS, first described by Nascimento in 2007, is characterized by a multinodular, infiltrating growth pattern with alternation of hypercellular and hypocellular myxoid areas [16]. Prominent curvilinear vessels are seen in the hypocellular areas. Tumour cells are arranged singly and in small clusters in the hypercellular areas, where they show epithelioid morphology. The epithelioid areas are generally admixed with areas of conventional MFS. Immunostains are negative for all markers [16].

Epithelioid gastrointestinal stromal tumour

Gastrointestinal stromal tumours (GISTs) are the most common mesenchymal neoplasms of the gastrointestinal tract and have been reported in all age groups. However, they occur predominantly in adults older than 50 years [17]. They typically exhibit a tan-white, fleshy cut-surface with foci of cystic degeneration, hemorrhage, or necrosis. Microscopically, most GISTs demonstrate three main histologic subtypes: spindle cell type, epithelioid type, and mixed spindle and epithelioid type [18]. Epithelioid GISTs account for approximately 20% of cases and are characterized by rounded cells arranged in nests or sheets, with variably eosinophilic to clear cytoplasm and vesicular nuclei. Approximately 10% of GISTs show a combination of both epithelioid and spindle cells [17].

Strong and diffuse immunoreactivity for KIT (CD117) is seen in about 95% of cases. Another common marker that is not as sensitive or specific for GIST is CD34 [18]. However, 5% of GISTs are negative for KIT by IHC and tend to be either KIT wild type or to harbour platelet-derived growth factor receptor alpha (PDGFRA) mutations [17]. These tumours often exhibit an epithelioid or mixed phenotype [18]. To improve the diagnostic accuracy for KIT-negative GISTs, newer markers like DOG1 has been studied [17,18].

Clear cell sarcoma

Clear cell sarcoma is an uncommon tumour, which typically arises in association with tendons and aponeuroses [19]. Histologically characterized by polygonal or spindle-shaped tumour cells with abundant eosinophilic or clear cytoplasm, vesicular nuclei, and prominent nucleoli [13]. Tumour cells are arranged in uniform, nested to the fascicular growth pattern. Melanin is rarely seen on H&E stains but can be detected by melanin stains in about 50% of cases. Mitosis is rare [13].

Clear cell sarcoma occasionally may be confused with epithelioid leiomyosarcoma, EMPNST, synovial sarcoma, and epithelioid sarcoma. Clear cell sarcoma shows positivity for HMB-45, neuron-specific enolase, and S-100. Cytokeratin, EMA, carcinoembryonic antigen, desmin, smooth muscle actin, and leukocyte common antigen are invariably absent [19]. Also known as melanoma of soft parts, it shares a similar immunohistochemical profile with malignant melanoma, however, it harbours a unique translocation t(12;22)(q13;q12) [13].

Monophasic synovial sarcoma

Synovial sarcomas are uncommon soft tissue tumours that usually occur in the extremities [20]. Two major histologic subtypes exist-monophasic and biphasic synovial sarcomas. The classical synovial sarcoma has a biphasic appearance with varying proportions of epithelial and spindle cells. The monophasic synovial sarcoma has a sarcomatous component. The spindle cells are positive for EMA, calretinin, cytokeratin, and vimentin, but negative for smooth muscle actin, S100, and CD117 ruling out leiomyosarcoma, MPNST, and GIST, respectively [20]. However, the gold standard to confirm the diagnosis of synovial sarcoma is to detect the t(X;18)(p11.2;q11.2) translocation [21].

Malignant perivascular epithelioid cell tumour (PEComa)

PEComa has been defined as “a mesenchymal tumour composed of histologically and immunohistochemically distinctive perivascular epithelioid cells” [22]. PEComas are considered ubiquitous tumours and have been described in various organs [22]. Tumour cells are arranged in sheets. The cytoplasm of the neoplastic cells varies from faintly eosinophilic to clear. Tumour cells can display considerable nuclear atypia, and necrosis can be present. Immunohistochemically, PEComa expresses myogenic and melanocytic markers, such as HMB45, Melan-A/Mart1, microphthalmia transcription factor (Mitf), actin and, less commonly, desmin [22]. Folpe et al. proposed criteria for the classification of these tumours as “benign”, “of uncertain malignant potential” and “malignant”. They observed a significant association between tumour size >5 cm, infiltrative growth pattern, high nuclear grade, necrosis, and mitotic activity >1/50 HPF and subsequent aggressive clinical behaviour of PEComas [23]. Morphologically, the differential diagnoses are clear cell sarcoma (positive for S100, which is usually negative in PEComa), epithelioid GIST (negative for melanocytic markers and positive for CD117), epithelioid leiomyosarcoma and alveolar soft part sarcoma (both negative for melanocytic markers) [24].

## Conclusions

A large group of sarcomas with an epithelioid morphology often mimics true epithelial neoplasms morphologically. A combination of clinical, morphological, immunohistochemical, and, when applicable, genetic findings help to arrive at a definitive diagnosis in most cases. In spite of diagnostic expertise and advances in the diagnostic modalities in the referral centres, some of these neoplasms remain elusive.
